# Technical efficiency in hip arthroscopy plateaus after approximately 240 cases while complication rates continue to decline: A 15‐year single‐surgeon series of 1490 consecutive procedures

**DOI:** 10.1002/jeo2.70959

**Published:** 2026-10-26

**Authors:** Andrea Mei, Leonardo Clausetti, Martial Metrop, Guillaume Girardot, Nicolas Bonin

**Affiliations:** ^1^ Università degli Studi di Milano, Scuola di Specializzazione in Ortopedia e Traumatologia Milano Italy; ^2^ Department of Orthopedic Surgery and Sport Medicine, Hospices Civils de Lyon, Hôpital de la Croix‐Rousse Lyon France; ^3^ Lyon‐Ortho‐Clinic Lyon France

**Keywords:** cumulative sum analysis, femoroacetabular impingement, hip arthroscopy, learning curve, segmented regression, traction time

## Abstract

**Purpose:**

To define the learning curve of a single surgeon performing hip arthroscopy using traction time as the primary metric of technical efficiency, and complication rate as a secondary endpoint, in a cohort large and long enough to model the inflection point with objective change‐point methods.

**Methods:**

A total of 1490 consecutive hip arthroscopies performed by one surgeon between January 2007 and April 2022 were retrospectively analysed. Cases were ordered chronologically and assigned a cumulative case number. Traction time was modelled against case number with Pearson correlation and a continuous two‐phase (broken‐stick) segmented regression; the breakpoint was estimated by grid‐search minimisation of the residual sum of squares, with a 95% confidence interval from non‐parametric bootstrap (400 resamples). A cumulative sum (CUSUM) chart was constructed. Experience phases were compared with the Welch *t* test, Mann–Whitney *U* test, and the two‐proportion test.

**Results:**

Traction time decreased with experience (*r* = −0.34; *p* < 0.001). Segmented regression identified a breakpoint at case 242 (95% confidence interval [CI], 208–258; model *R* ^2^ = 0.35): a first‐phase decline of 1.6 min per 10 cases was followed by a plateau near 35 min; the CUSUM peak (case 224) corroborated this transition. Mean traction time fell from 54.8 to 35.6 min before versus after the breakpoint (*p* < 0.001). Graded with the validated modified Clavien–Dindo classification for hip preservation surgery, the patient‐level complication rate decreased across consecutive phases from 14.7% (cases 1–150) to 3.1% (151–500) to 1.5% (501–1490), and continued to decline beyond the efficiency plateau; most complications were Grade I.

**Conclusions:**

In one of the largest single‐surgeon series reported, technical efficiency (traction time) stabilised at approximately 242 cases, whereas safety continued to improve well beyond this point. In this single‐surgeon experience, the learning curve extended over several hundred cases, well beyond the 20–30 cases reported in early small series; whether these thresholds apply to other surgeons and training contexts requires confirmation.

**Level of Evidence:**

Level IV, therapeutic case series.

## Graphical abstract

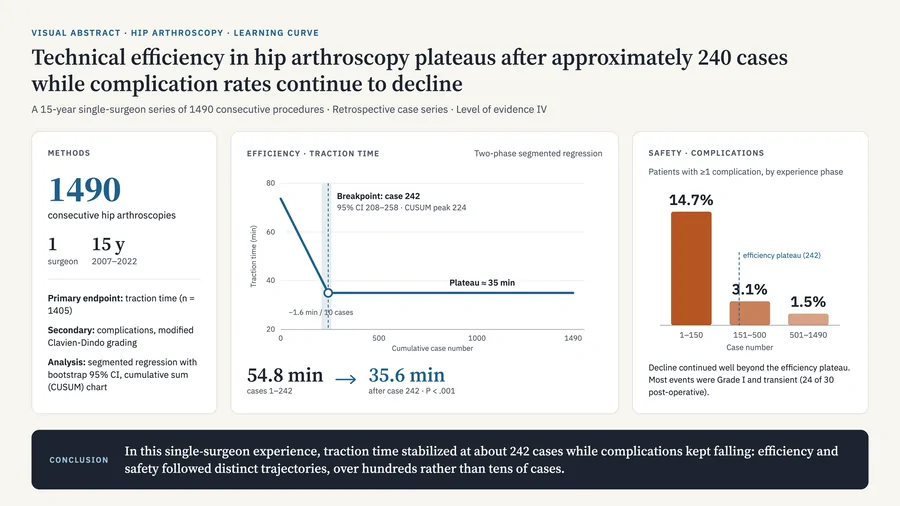

**Abbreviations**
CI: confidence interval
CUSUM: cumulative sum
FAI: femoroacetabular impingement
RSS: residual sum of squares

## INTRODUCTION

Hip arthroscopy has expanded over the past two decades to become the treatment of choice for femoroacetabular impingement (FAI) and associated labral and chondral pathology, with utilisation rising several‐fold within a single decade [15]. It is consistently described as one of the most technically demanding procedures in orthopaedic surgery, requiring advanced spatial skills, specialised instrumentation and multiple technical checkpoints from patient positioning to capsular closure [6], with a learning curve characterised as ‘steep’ [5, 9]. Yet the number of cases required to reach proficiency remains controversial: published estimates range from as few as 20–30 cases in early small series to more than 500 cases in large administrative‐database analyses [5, 9, 14]. This wide spread reflects heterogeneity in the metric chosen to define competence (operative or traction time, complications, reoperation, patient‐reported outcomes), in the statistical method used, and in surgeon and case‐mix profiles [1, 3, 5]. Recent pooled and national registry analyses have confirmed that surgeon experience influences operative efficiency and outcomes, although the magnitude of the effect and the experience thresholds involved remain uncertain [8, 16].

Single‐surgeon series are particularly informative because they isolate individual skill acquisition from the inter‐surgeon variability that confounds multi‐surgeon and registry studies. However, the available single‐operator cohorts are small and short, typically comprising 40–225 cases observed over a few years [4, 10, 13, 21]. Few studies have applied objective change‐point methodology—such as cumulative sum (CUSUM) analysis or segmented regression—to locate the inflection point of the curve rather than imposing an arbitrary cutoff [5, 13].

A 15‐year consecutive series of 1490 hip arthroscopies performed by a single surgeon was therefore analysed. The purpose of this study was to define the learning curve using traction time as the primary metric of technical efficiency, with complication rate as a secondary endpoint, and to estimate the inflection point with segmented regression (with a bootstrap confidence interval) and CUSUM analysis. It was hypothesised that technical efficiency would plateau after a substantially larger number of cases than the commonly cited 30, and that safety would continue to improve beyond the efficiency plateau.

## METHODS

This was a retrospective analysis of a single‐surgeon database prospectively maintained at one institution. All 1490 consecutive hip arthroscopies with a documented operative date, performed between 02 January 2007 and 19 April 2022, were included; there were no exclusions based on indication or concomitant procedure. Cases were ordered chronologically by operative date, and each case was assigned a cumulative case number reflecting the surgeon's accumulated experience at the time of surgery. The predominant indication was FAI (cam, pincer and mixed types), followed by pre‐osteoarthritis, synovial chondromatosis, isolated labral tears, and instability. Institutional review board approval and the requirement for informed consent are addressed in the title page.

The primary endpoint was traction time (minutes), a well‐established surrogate of technical efficiency in hip arthroscopy [5]. Secondary endpoints were intra‐operative incidents (technical adverse events such as instrument breakage or iatrogenic chondrolabral injury), post‐operative complications (predominantly traction‐related: perineal or cutaneous pressure ulcer, transient paraesthesia, transient erectile dysfunction consistent with pudendal neurapraxia, and heterotopic ossification), and reoperation. Complications were ascertained from the operative and follow‐up records. Post‐operative complications were graded with the validated modified Clavien‐Dindo classification for hip preservation surgery [18], which assigns five grades according to the treatment required and the potential for long‐term morbidity (Grade I, no treatment required; Grade II, pharmacological/non‐operative treatment; Grade III, intervention without [IIIa] or under [IIIb] general anaesthesia; Grade IV, life‐threatening complication or permanent disability; Grade V, death). The same scheme has been applied prospectively to a large multicentre hip‐arthroscopy cohort [12]. Intra‐operative events (instrument breakage, suture‐anchor failure, iatrogenic chondral/labral injury) were recorded and reported together with the graded post‐operative complications.

### Statistical analysis

Continuous variables are reported as mean ± standard deviation and median (range); categorical variables as counts and percentages. Traction time was unavailable in 85 of the 1490 procedures: it was not documented in 66 cases and was recorded as zero, and therefore considered implausible, in 19 cases. These 85 cases were treated as missing, leaving 1405 cases for the traction analysis; they were retained in the complication analysis. The association between cumulative case number and traction time was assessed with the Pearson correlation coefficient and modelled with a continuous two‐phase (broken‐stick) segmented linear regression sharing a common knot; the breakpoint was estimated by grid‐search minimisation of the residual sum of squares across candidate case numbers, and its 95% confidence interval (CIs) was obtained by non‐parametric bootstrap (400 resamples). A CUSUM chart of the cumulative deviation of each case from the cohort mean was constructed to provide an independent, model‐free localisation of the transition point [13]. The early (pre‐breakpoint) and late (post‐breakpoint) phases, and the first 100 versus subsequent cases, were compared with the Welch *t*‐test (means) and the Mann–Whitney *U* test (medians). Complication proportions across consecutive experience phases were compared with the two‐proportion test. Statistical significance was set at a two‐sided alpha of 0.05.

## RESULTS

The cohort comprised 1490 consecutive procedures spanning 15 years (2007–2022). Mean age at surgery was 33.0 ± 10.9 years (Table 1). Annual volume rose from fewer than 75 cases in the early years to more than 100 cases per year from 2013 onward (Table 2).

**Table 1:** Cohort characteristics.

Variable	Value
Procedures, *n.*	1490
Study period	January 2007 to April 2022 (15 years)
Age at surgery, years	33.0 ± 10.9
Predominant indication	Femoroacetabular impingement (cam/pincer/mixed)
Traction time analysed, *n*	1405
Patients with ≥ 1 complication	3.2% (48/1490)

**Table 2:** Annual case volume and mean traction time.

Year	Cases, *n*	Mean traction, min	Median, min
2007	7	70.0	75
2008	42	71.7	75
2009	29	62.1	60
2010	38	59.5	60
2011	53	51.9	50
2012	75	40.1	40
2013	119	34.7	35
2014	144	33.4	30
2015	101	35.8	35
2016	110	38.2	38
2017	113	36.7	35
2018	116	37.2	35
2019	132	37.8	35
2020	119	32.2	30
2021	162	36.1	35
2022 (4 months)	45	32.8	30

Traction time (*n* = 1405) averaged 38.9 ± 14.6 min (median 35; range 5–100) and correlated negatively with cumulative case number (*r* = −0.34; *p* < 0.001). Segmented regression identified a breakpoint at case 242 (95% bootstrap CI, 208–258; model *R* ^2^ = 0.35; Figure 1). The first‐phase slope was −0.160 min per case (approximately 1.6 min per 10 cases), whereas the second‐phase slope was effectively zero, defining a plateau near 35 min. The CUSUM chart peaked at case 224 (Figure 2), in close agreement with the regression breakpoint.

**Figure 1:**
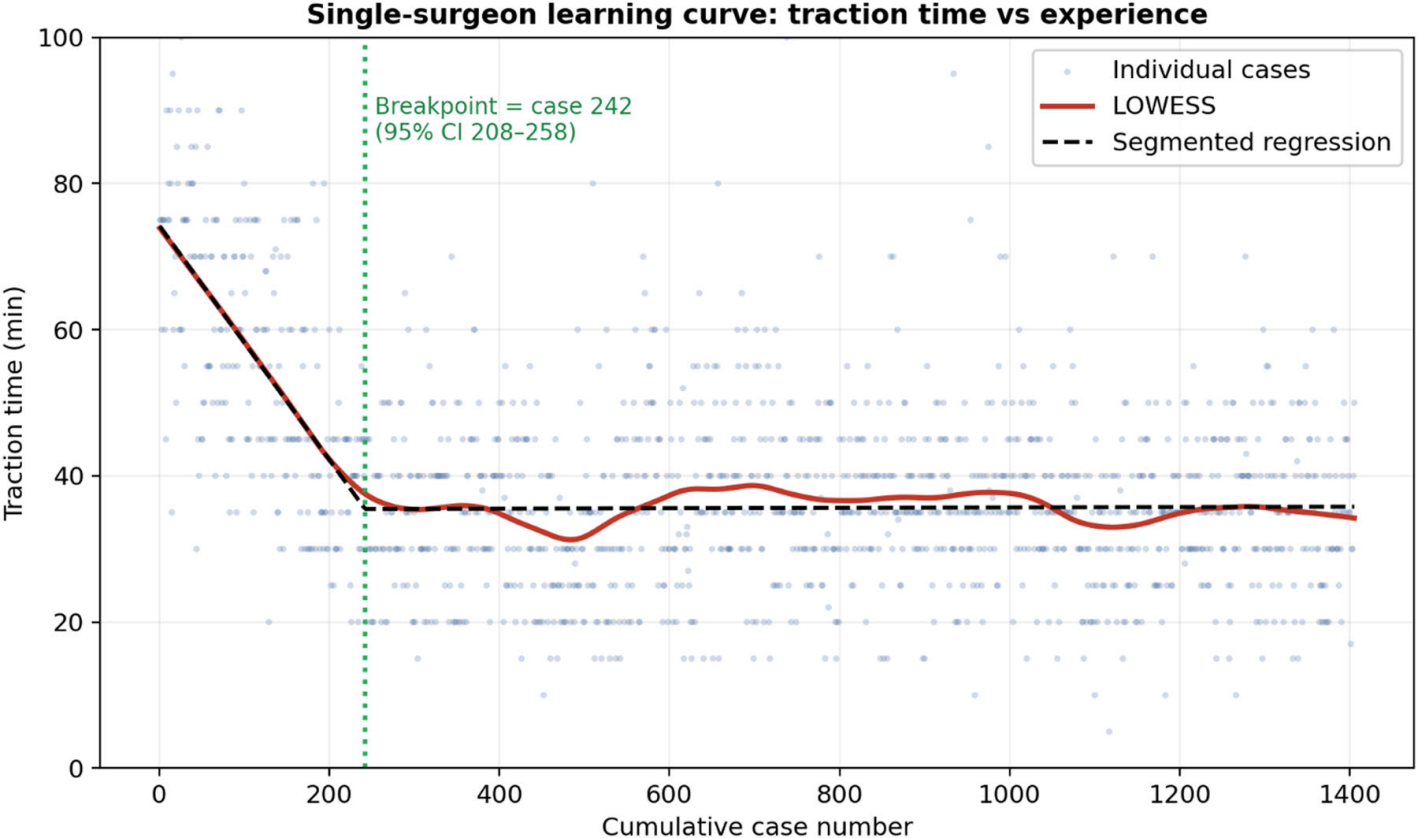
Traction time versus cumulative case number. The red line is a LOWESS smoother and the dashed black line the two‐phase segmented regression; the green line marks the breakpoint at case 242 (95% bootstrap confidence interval [CI], 208–258).

**Figure 2:**
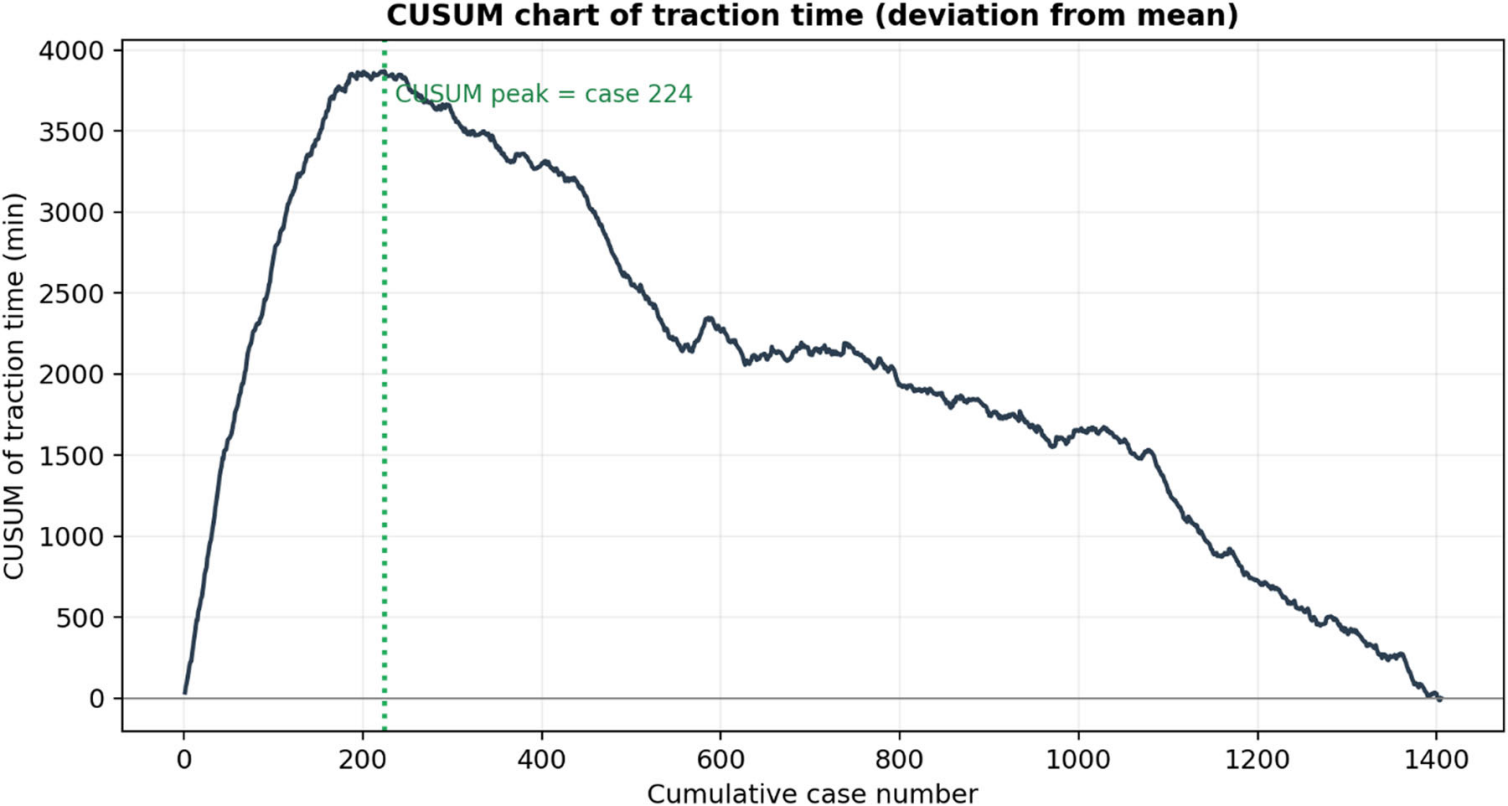
Cumulative sum (CUSUM) chart of traction time (cumulative deviation from the cohort mean). The peak at case 224 marks the transition from above‐average to below‐average operative duration, corroborating the regression breakpoint.

Mean traction time was 54.8 min for cases 1–242 versus 35.6 min thereafter (difference 19.2 min; *p* < 0.001), and 66.2 versus 36.8 min for the first 100 versus subsequent cases (*p* < 0.001) (Table 3). The annual mean traction time fell steeply from 75 min (2007–2008) to approximately 33–35 min by 2013 and remained stable thereafter (Table 2; Figure 3).

**Table 3:** Traction time by experience phase.

Comparison	Early	Late	*p*
Cases 1–242 versus >242	54.8 min (*n* = 242) (AUTHORS TO VERIFY *n*: must match case‐numbering definition in statistical analysis)	35.6 min (*n* = 1163)	<0.001
Median (Mann–Whitney)	55 min	35 min	<0.001
First 100 versus subsequent	66.2 min (*n* = 100)	36.8 min (*n* = 1305)	<0.001

**Figure 3:**
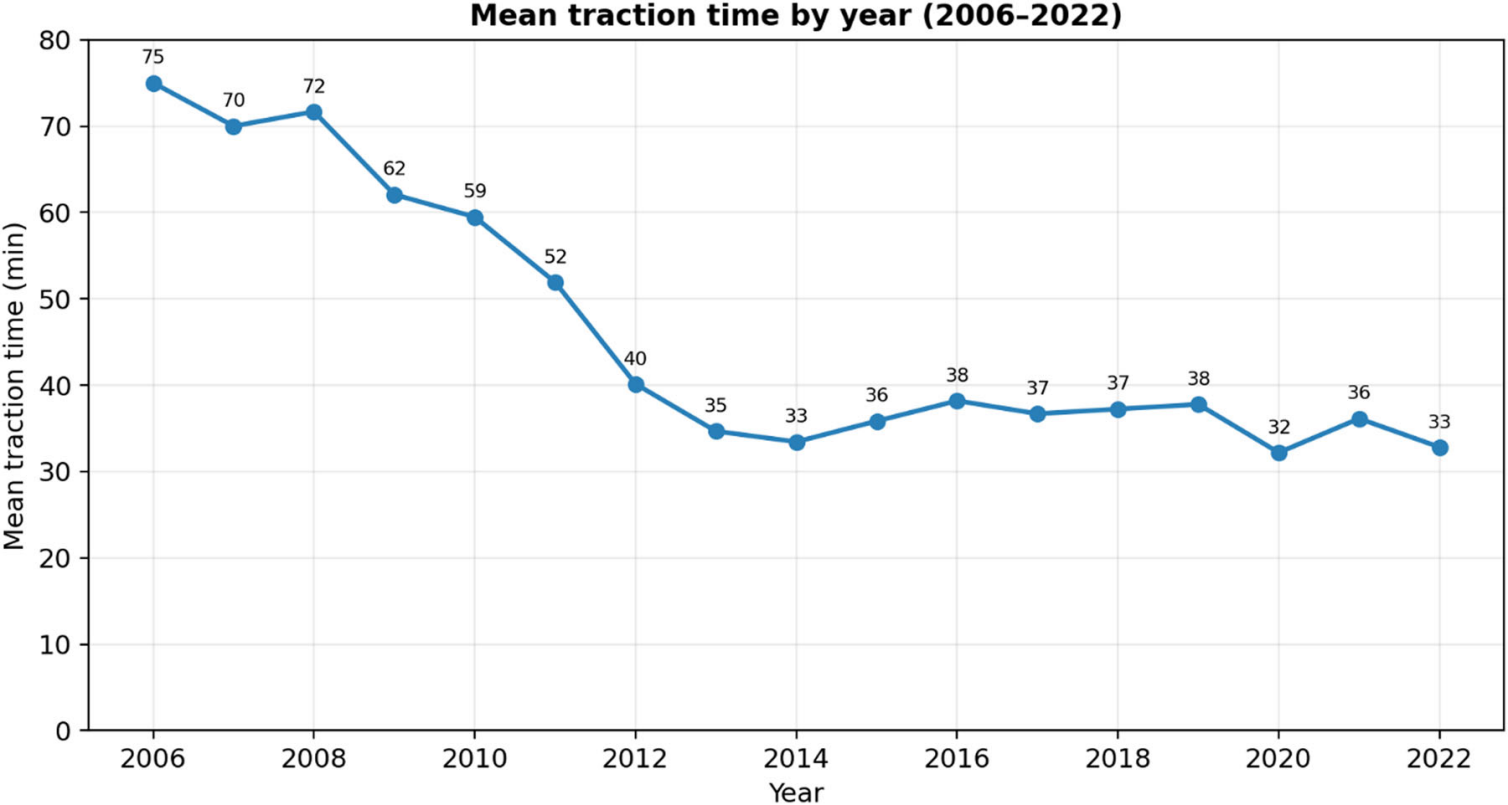
Mean traction time by calendar year across the series, showing a steep decline through 2012–2013 followed by a stable plateau.

Across the cohort there were 21 intra‐operative events (instrument breakage, *n* = 8; iatrogenic chondral/labral injury, *n* = 7; suture‐anchor failure, *n* = 4; suboptimal labral repair, *n* = 2) and 30 post‐operative complications, affecting 48 of 1490 patients overall (3.2%). Graded with the modified Clavien–Dindo classification [12, 18], post‐operative complications were Grade I in 24 cases (predominantly traction‐related pressure ulcer, transient pudendal or lateral‐femoral‐cutaneous neurapraxia, and asymptomatic heterotopic ossification), Grade IIIb in 5 cases (revision arthroscopy, psoas release, or open complement), and Grade IV in 1 case (femoral head osteonecrosis); there were no Grade II, IIIa or V complications (Table 4).

**Table 4:** Complications graded by the modified Clavien–Dindo classification for hip preservation surgery, and by experience phase.

Category	*n*
Intra‐operative events	21
Instrument breakage	8
Iatrogenic chondral/labral injury	7
Suture‐anchor failure	4
Suboptimal labral repair	2
Post‐operative complications	30
Grade I	24
Grade II	0
Grade IIIa	0
Grade IIIb	5
Grade IV	1
Grade V	0
*Patients with ≥ 1 complication, by experience phase:*	

Phase (case numbers)	**Cases, *n***	**Patients with complication, *n***	**Rate (%)**
1–150	150	22	14.7
151–500	350	11	3.1
501–1490	990	15	1.5
Overall	1490	48	3.2

The patient‐level complication rate decreased monotonically across consecutive experience phases: 14.7% (cases 1–150), 3.1% (151–500) and 1.5% (501–1490) (Figure 4). In a direct comparison, the rate was 20% in the first 100 cases versus 2.0% thereafter (*p* < 0.001). Notably, the complication rate continued to fall well beyond the case‐242 efficiency plateau.

**Figure 4:**
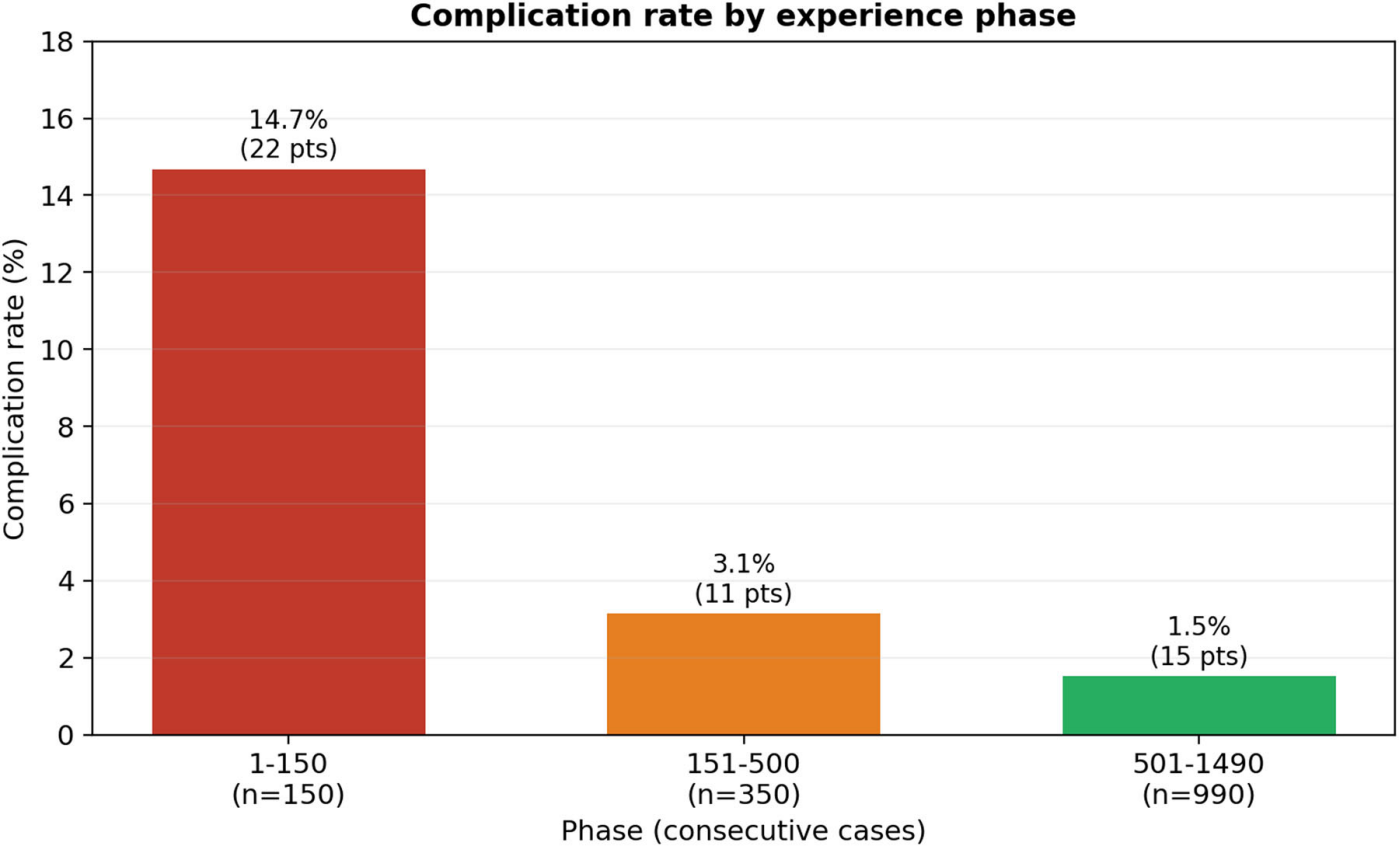
Patient‐level complication rate by consecutive experience phase, decreasing progressively from 14.7% to 1.5%.

## DISCUSSION

The most important finding of this study is that, in a single‐surgeon cohort of large size, the technical‐efficiency component of the learning curve—traction time— stabilised at approximately 242 cases, with a narrow bootstrap confidence interval (208–258) and concordant localisation by an independent CUSUM analysis. This estimate is an order of magnitude higher than the 20–30 cases frequently cited in early small series [5, 9, 13], but corroborates the conclusion of the largest study conducted on the subject, involving 8041 hip arthroscopies, in which Mehta et al. [14] found the learning curve to be ‘surprisingly demanding’, with surgeon career‐volume strata of 0–97 (low), 98–388 (medium), 389–518 (high) and ≥519 (very high) cases; the breakpoint of 242 observed here falls squarely within their medium‐volume band and far above the small‐series thresholds. The present series is among the largest single‐surgeon series reported and is one of the few to combine segmented regression with a bootstrap confidence interval and CUSUM corroboration, rather than imposing an arbitrary cutoff. In a recent single‐surgeon series of 71 procedures for FAI published in this journal, case sequence correlated strongly with both surgical and traction time, and 30‐day complications were more frequent in the earliest group; [21] the present series extends these observations to a cohort approximately twenty times larger and locates the inflection point objectively.

A second, clinically reassuring finding is the decoupling of efficiency and safety. Whereas traction time plateaued at case 242, the complication rate continued to decline across the entire series, from 14.7% in the first 150 cases to 1.5% after case 500. The same dissociation is implicit in the registry data of Mehta et al. [14], in which the steep fall in reoperation risk appeared only in the very‐high‐volume stratum (≥519 cases), well beyond the threshold at which operative metrics generally plateau. Together, these observations suggest that ‘proficiency’ is not a single threshold: technical fluency is acquired comparatively early, while the optimisation of judgment, indication, and complication avoidance is a longer, continuing process. This is consistent with smaller single‐surgeon series reporting operative‐time plateaus at 75–100 cases [4, 10] and early‐phase excess complications [7, 21], and with the view that the term ‘steep learning curve’ conflates several distinct dimensions of skill [1, 3]. Reassuringly, when graded with the validated modified Clavien–Dindo scheme [18], the overwhelming majority of post‐operative complications in this series were Grade I and transient, a distribution closely mirroring the largest prospectively graded hip‐arthroscopy series, in which 7.2% of hips had a Grade I complication and higher grades were rare [12]. That series also linked traction time longer than 60 min to a significantly higher complication rate and to pudendal neurapraxia [12], underscoring why the marked early‐phase reduction in traction time observed here is not merely a measure of efficiency but is itself plausibly protective.

These findings should be interpreted alongside the most recent pooled and registry evidence. A 2026 meta‐analysis of 18 studies (2624 procedures) comparing early and late experience phases found significantly shorter operative and traction times in late cases, with a pooled reduction in traction time of approximately 16 min, comparable to the 19‐min difference observed here, whereas safety outcomes were largely unchanged [16]. The apparent discrepancy with the continued decline in complications observed in the present series may reflect the limited size of the pooled cohorts, their arbitrary early/late cutoffs and heterogeneous complication definitions; the present series alone corresponds to more than half of the procedures pooled in that meta‐analysis and applies a validated grading scheme across the entire experience. Similarly, a national registry study found that cumulative surgeon volume was associated with improved patient‐reported outcomes after hip arthroscopy, although the magnitude of the effect was small [8], and a recent umbrella review critically appraised the methodological reliability of hip arthroscopy meta‐analyses published between 2020 and 2025 [17]. Together, these data support the need for large, consecutive, well‐characterised series to complement pooled estimates.

A distinctive strength of this cohort is its 15‐year span. Over this period hip arthroscopy itself evolved profoundly—from predominantly resective procedures (labral debridement, limited osteoplasty) toward labral preservation and repair, routine cam and pincer correction, and capsular management including capsular closure [6]. The learning curve described here therefore reflects not only the consolidation of one surgeon's individual skill but also its co‐evolution with two decades of scientific and technical progress in the discipline. That a stable traction‐time plateau persisted despite the progressive adoption of these more complex and time‐consuming techniques argues for genuine skill consolidation rather than mere simplification of the case‐mix. The independent influence of this two‐decade technical evolution on operative metrics and outcomes is substantial and merits dedicated analysis.

These figures should also be read in their historical context. Over the study period, hip arthroscopy underwent rapid growth, yet the procedure long remained concentrated in the hands of a small number of high‐volume pioneers, with only a minority of surgeons performing more than a handful of cases per year [15]. Structured training in hip arthroscopy was largely unavailable when this series began, which may partly explain the length of the learning curve observed here. The subsequent development of dedicated fellowships, cadaveric laboratories, structured mentorship, and validated virtual‐reality simulators [2, 20] is expected to shorten the curve for surgeons trained today, so that the several‐hundred‐case threshold described here should be interpreted as characteristic of an era of largely self‐taught adoption rather than as an immutable property of the procedure.

These findings may be of practical interest. Benchmarks derived from objective change‐point analysis, rather than from arbitrary case counts, may help set realistic expectations for surgeons adopting hip arthroscopy and may inform discussions on mentored early experience and on case‐load targets in fellowship training and simulation curricula [7, 19, 20]. The high early‐phase complication rate in this and other series [7, 21] is consistent with the value of mentorship and graduated case complexity during the initial experience; however, the optimal structure of such pathways cannot be defined on the basis of a single‐surgeon series.

### Strengths and limitations

The principal strengths are the cohort size, the uninterrupted 15‐year single‐operator continuity, and the use of objective change‐point methods with a bootstrap confidence interval and independent CUSUM corroboration. Several limitations apply. First, this is a retrospective, single‐centre, single‐surgeon case series (Level IV); although this design optimally isolates individual skill, it limits generalisability, and the thresholds reported here should not be transferred directly to other surgeons or training settings. Second, traction time is a surrogate of efficiency that is also influenced by case complexity, which increased over time. Third, complications and reoperations after hip arthroscopy are also influenced by patient‐related and postoperative factors independent of surgeon experience, such as postoperative falls [11], which could not be systematically captured. Fourth, follow‐up patient‐reported outcomes were available for only a small, predominantly early subset of the cohort (paired scores in roughly 8% of patients, collected mainly before 2015) and did not vary with surgeon experience; they were therefore not analysed as a learning‐curve endpoint, and the absence of systematic follow‐up scores in the later cohort remains a limitation.

## CONCLUSIONS

In one of the largest single‐surgeon hip arthroscopy series reported to date, technical efficiency defined by traction‐time stabilisation was reached at approximately 242 cases, whereas complication rates continued to decline well beyond this point. In this experience, the learning curve was measured in hundreds rather than tens of cases, and efficiency and safety followed distinct trajectories. Whether these thresholds apply to surgeons trained in contemporary structured programmes remains to be established.

## AUTHOR CONTRIBUTIONS

**Andrea Mei**: Conception and design; data analysis; drafting. **Leonardo Clausetti**: Data collection; drafting; critical revision. **Martial Metrop** and **Guillaume Girardot**: Data collection and interpretation; critical revision. **Nicolas Bonin**: Study supervision; provision and curation of the surgical database; critical revision for important intellectual content. All authors approved the final version.

## FUNDING INFORMATION

The authors have no funding to report.

## CONFLICT OF INTEREST STATEMENT

Nicolas Bonin reports activities outside the submitted work (consultancy/royalties) that the authors will declare in full at submission. The remaining authors declare no conflicts of interest.

## ETHICS STATEMENT

This study is a retrospective analysis of a prospectively maintained institutional surgical database for that reason formal approval was not required under local regulations. No individually identifiable patient data or images are reported.

## Data Availability

The data supporting the findings of this study are available from the corresponding author upon reasonable request; individual patient records are not publicly available for reasons of confidentiality.
